# Supplementary material for: Autophagy drives the conversion of developmental neural stem cells to the adult quiescent state
Source: Nat Commun. 2023 Nov 24;14:7541. doi: 10.1038/s41467-023-43222-1 (PMC10673888; doi:10.1038/s41467-023-43222-1)
Supplement: Supplementary file 1 — Supplementary Information [file 41467_2023_43222_MOESM1_ESM.pdf]

SUPPLEMENTARY INFORMATION

Autophagy drives the conversion of developmental neural stem cells to the adult quiescent state

Isabel Calatayud-Baselga, Lucía Casares-Crespo, Carmina Franch-Ibáñez, José Guijarro-Nuez, Pascual Sanz, Helena Mira

- Supplementary Figures

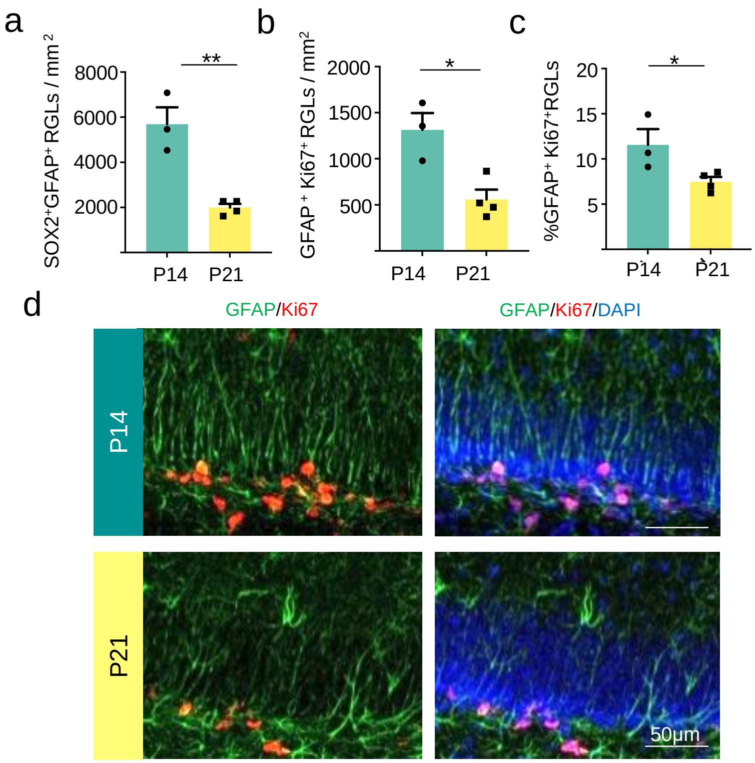

Supplementary Figure 1. Dentate gyrus P14 RGLs continue to enter quiescence until P21. a. Quantification of RGLs (GFAP$^+$/SOX2$^+$)/mm^2 in the dentate gyrus of postnatal 14 (P14) and 21 (P21) days old wild type mice. **b.** Quantification of active RGLs (Ki67$^+$/GFAP$^+$)/mm2 in P14 and P21 mice. **c.** Percentage of active RGLs in P14 and P21 mice. Data are represented as mean ± SEM from n=3 (P14) or n=4 (P21) mice. Statistics of a, b, c: Unpaired t-test, two-tailed. **d.** Representative immunohistochemistry confocal images of RGLs labelled for GFAP (green) and Ki67 (red). Scale bar: 50 µm. p-value: *<0.05; **<0.001. Source data are provided as a Source Data file. RGLs, Radial Glia-Like Cells. P14, Postnatal day 14. P21, Postnatal day 21.

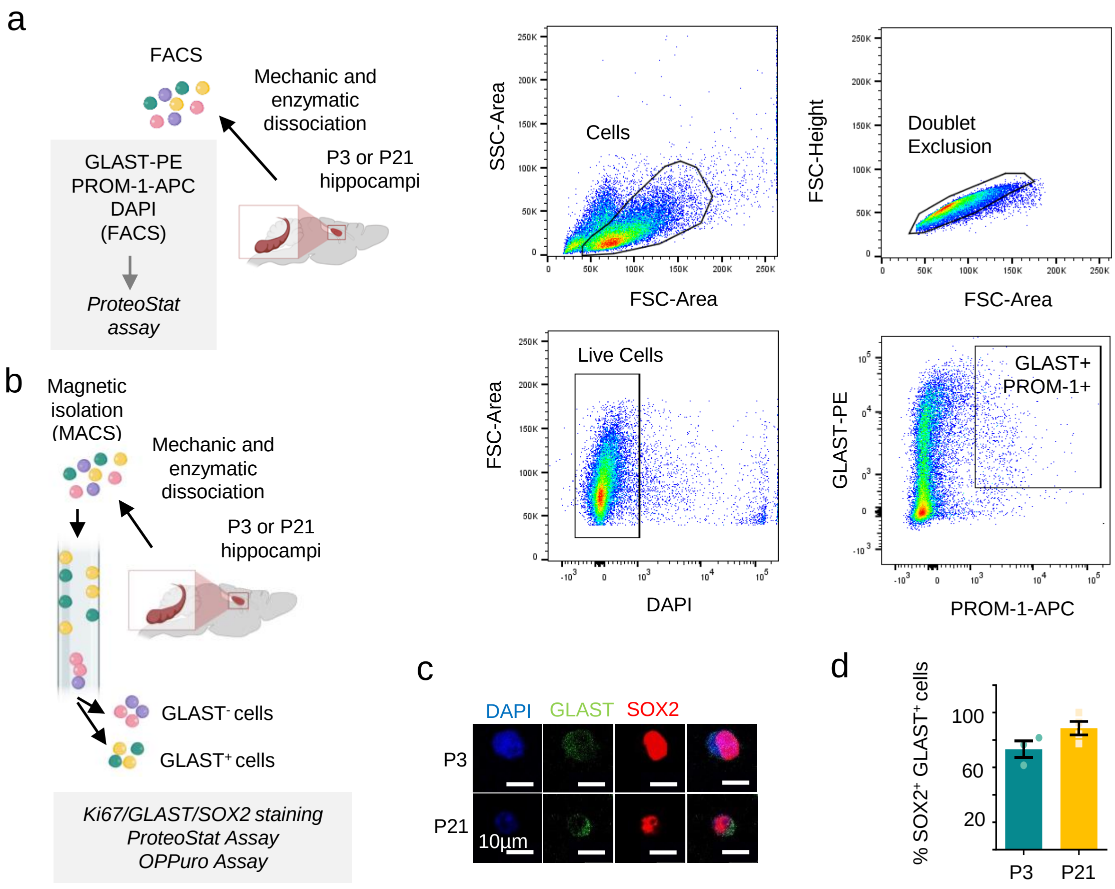

Supplementary Figure 2. NSC isolation and characterization by FACS and MACS. a. (Left) Schematic representation of NSCs isolation by FACS from postnatal 3 (P3) and 21 (P21) day old mice hippocampi using GLAST-PE and PROM-1-APC antibodies. (Right) Representative images of FACS gating strategy (P3 mice). Forward scatter/side scatter gatings were used to remove doublets and debris. DAPI negative cells were considered viable. NSCs were isolated based on GLAST-PE and PROM-1-APC positive expression. NSCs sorted from P3 and P21 mice were 2.16 ± 0.53 and 0.54 ± 0.13% (mean ± SEM), respectively, of the total cells analyzed. **b.** Schematic representation of magnetic GLAST$^+$ cells isolation from postnatal 3 (P3) and 21 (P21) days old mice hippocampi using MACS (Miltenyi Biotec) disaggregation and isolation kit. **c.** Representative immunocytochemestry images of GLAST/SOX2 in GLAST$^+$ cells isolated of postnatal 3 and 21-day-old mice. Scale bar: 10 µm. **d.** Percentage of SOX2$^+$GLAST$^+$ cells isolated from P3 and P21 mice. The experiment was repeated 3 times with similar results. Data are represented as mean ± SEM from n=3 independent isolations of 8 P3 mice and n=4 independent isolations of 6 P21 mice. Statistics: unpaired t-test, two-tailed. Both representations in a. and b. were created with BioRender.com. Source data are provided as a Source Data file. FACS, Fluorescent Activated Cell Sorter. MACS, Magnetic Activated Cell Sorter. P3, Postnatal day 3. P21, Postnatal day 21.

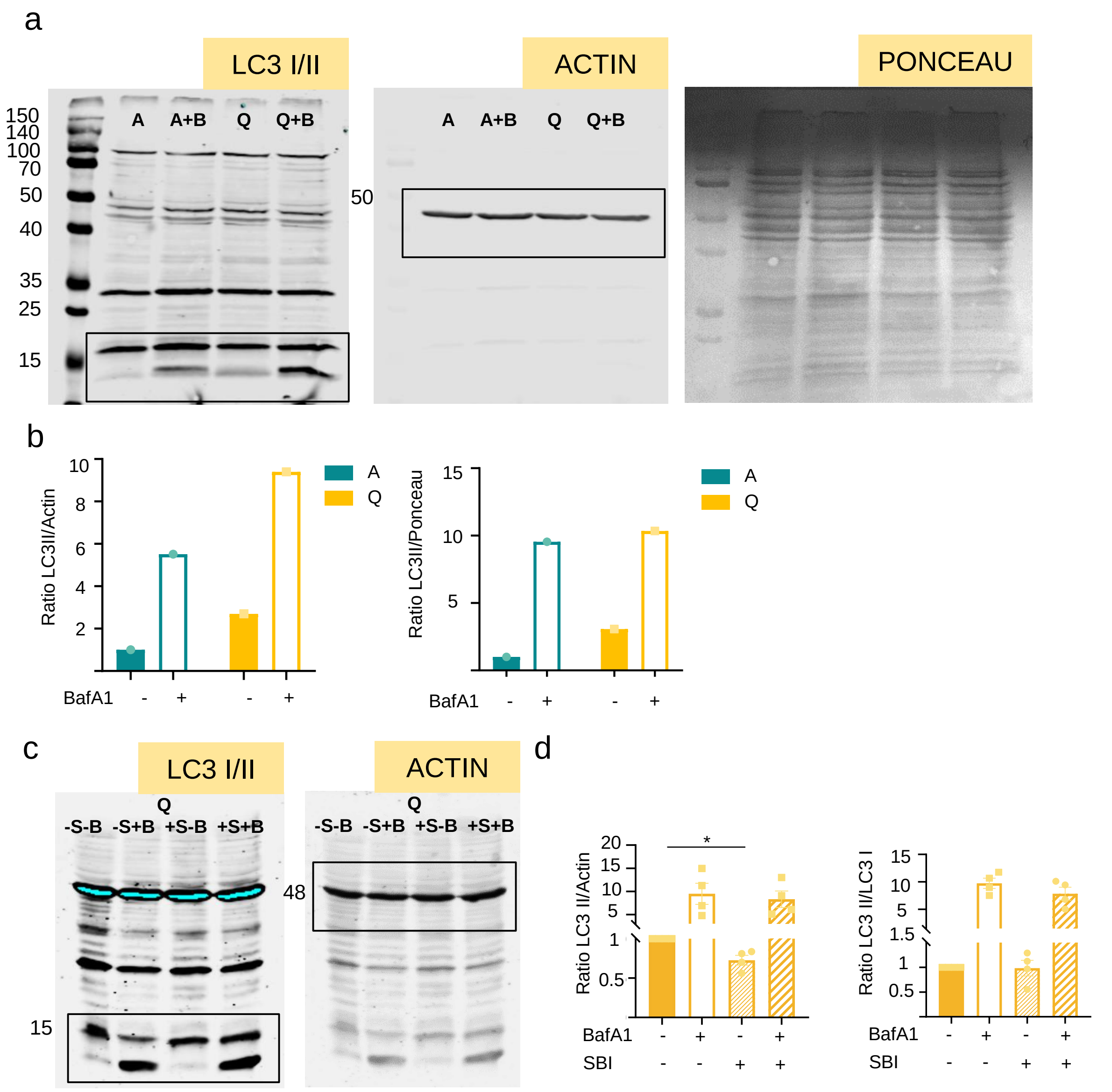

Supplementary Figure 3. Autophagy marker LC3 II accumulates in Q-NSPCs. a. Original full-length Western immunoblots showing the cropped region used to compose the final image displayed in Figure 2f. **b.** Quantification of LC3 II immunoblot in A-NSPCs and Q-NSPCs treated with BafA1 during 6h. β-Actin (left) and Ponceau (right) were used as a loading control to normalize LC3 II levels, respectively. **c.** Western immunoblots of LC3 I and LC3 II in Q-NSPCs treated for 1 h with SBI and 6 h with BafA1. β-Actin was used as a loading control. **d.** Quantification of LC3 II immunoblot in Q-NSPCs treated for 1 h with SBI and 6 h with BafA1 relative to β-Actin levels (left) or to LC3 I (right). The LC3 II / LC3 I ratio accounts for the degree of LC3 lipidation. The experiment was repeated 4 times with similar results. Data are represented as mean ± SEM from n=4 cultures (Fig. d). In Fig. b n=1 culture is represented. Statistics: one-sample or paired t-test, two tailed. p-value: *<0.05. Source data and all blots are provided as a Source Data file. A, Active NSPCs. Q, Quiescent NSPCs. S, SBI. B, BafA1. BafA1, Bafilomycin A1.

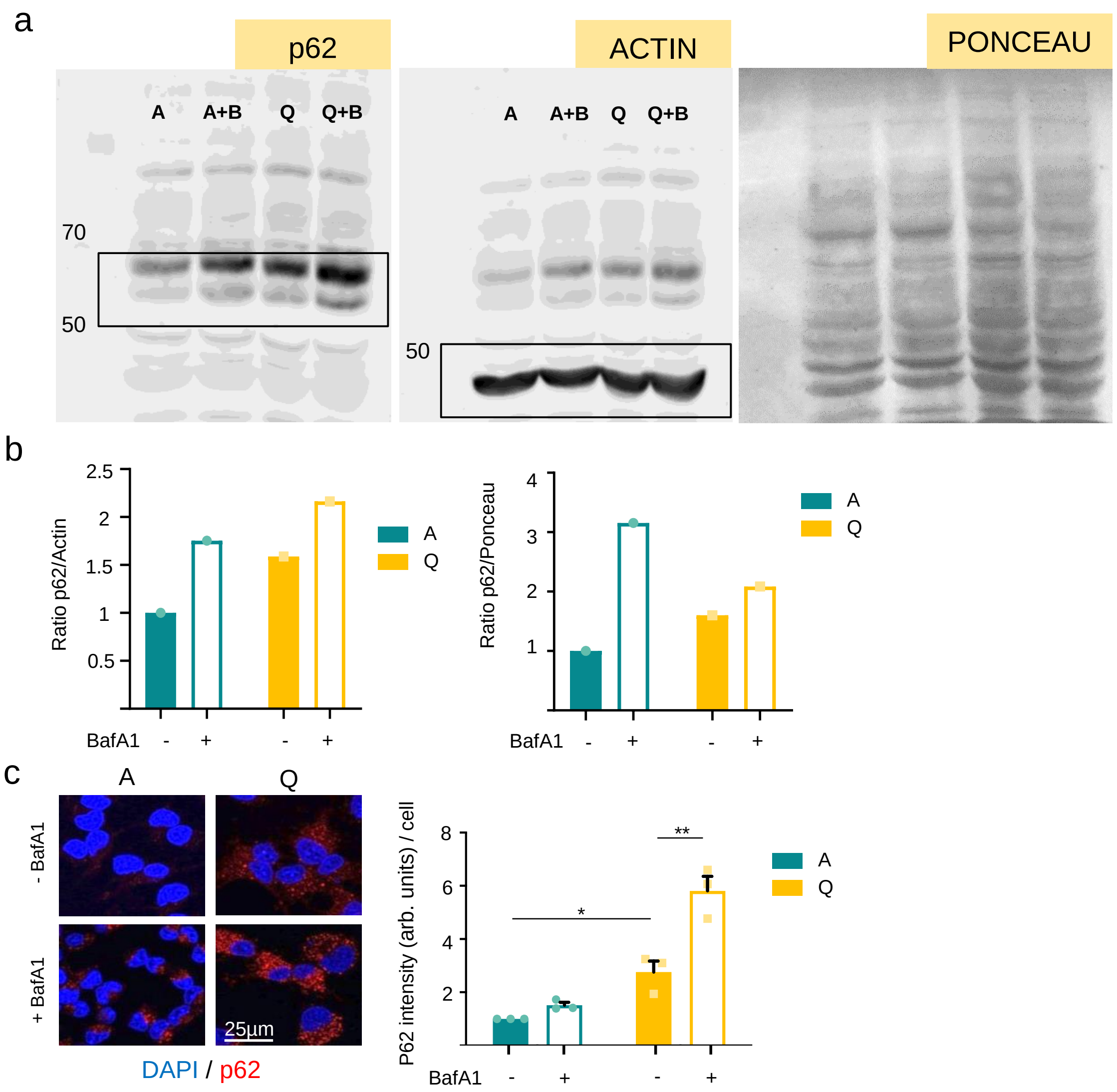

Supplementary Figure 4. Autophagy marker SQSTM/p62 accumulates in Q-NSPCs. a. Original full-length Western immunoblots showing the cropped region used to compose the final image displayed in Figure 2k. **b.** Quantification of p62 immunoblot in A-NSPCs and Q-NSPCs treated with BafA1 during 6h. β-Actin (left) and Ponceau (right) were used as a loading control to normalize p62 levels, respectively. **c.** (Left) Representative immunofluorescence confocal images of p62 label (red) in Q-NSPC and A-NSPC cultures treated with BafA1. Scale bar: 25 µm.(Right) Quantification of p62 protein level in A-NSPCs and Q-NSPCs treated with BafA1 during 6h. measured as fluorescence intensity. The experiment was repeated 3 times with similar results. Data are represented as mean ± SEM from n=3 cultures (Fig. c). In Fig. b n=1 culture is represented. At least 15 cells were analyzed per experiment and condition. Statistics: two-way ANOVA. p-value: *<0.05; **<0.001. Source data and all blots are provided as a Source Data file. A, Active NSPCs. Q, Quiescent NSPCs. B, BafA1. BafA1, Bafilomycin A1.

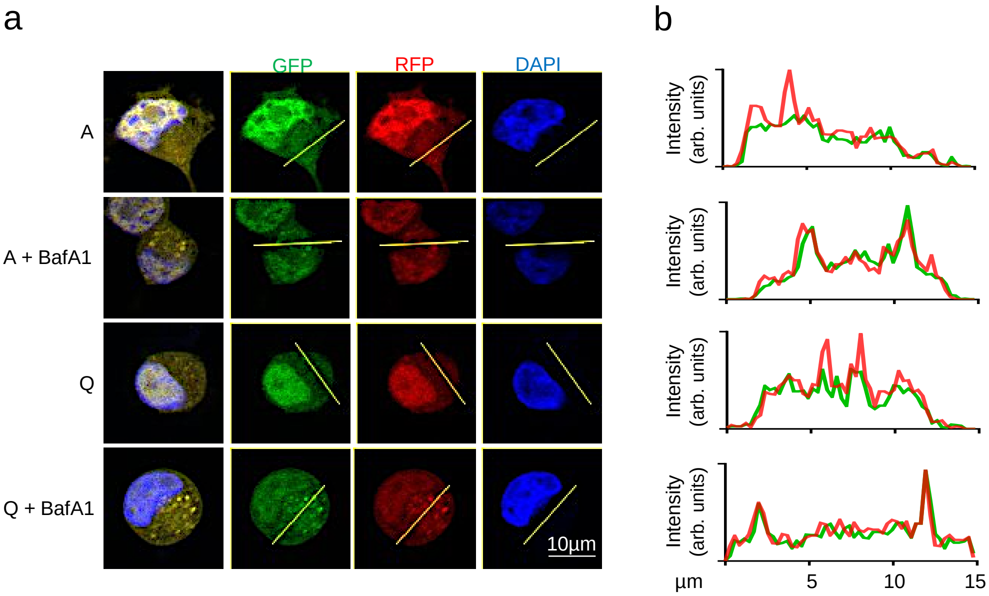

Supplementary Figure 5. NSPC electroporation and pRFP-GFP-LC3 tandem sensor analysis. a. Representative immunofluorescence confocal images of A-NSPC and Q-NSPC cultures electroporated with pRFP-GFP-LC3 plasmid. Cultures were treated with 100nM Bafilomycin A1 for 6h. Scale bar: 10 µm. **b.** Fluorescence intensity histogram corresponding to the section plotted on the confocal images. Green and red peaks correspond to autophagosomes, and red only peaks correspond to autolysosomes. A, Active NSPCs. Q, Quiescent NSPCs. BafA1, Bafilomycin A1.

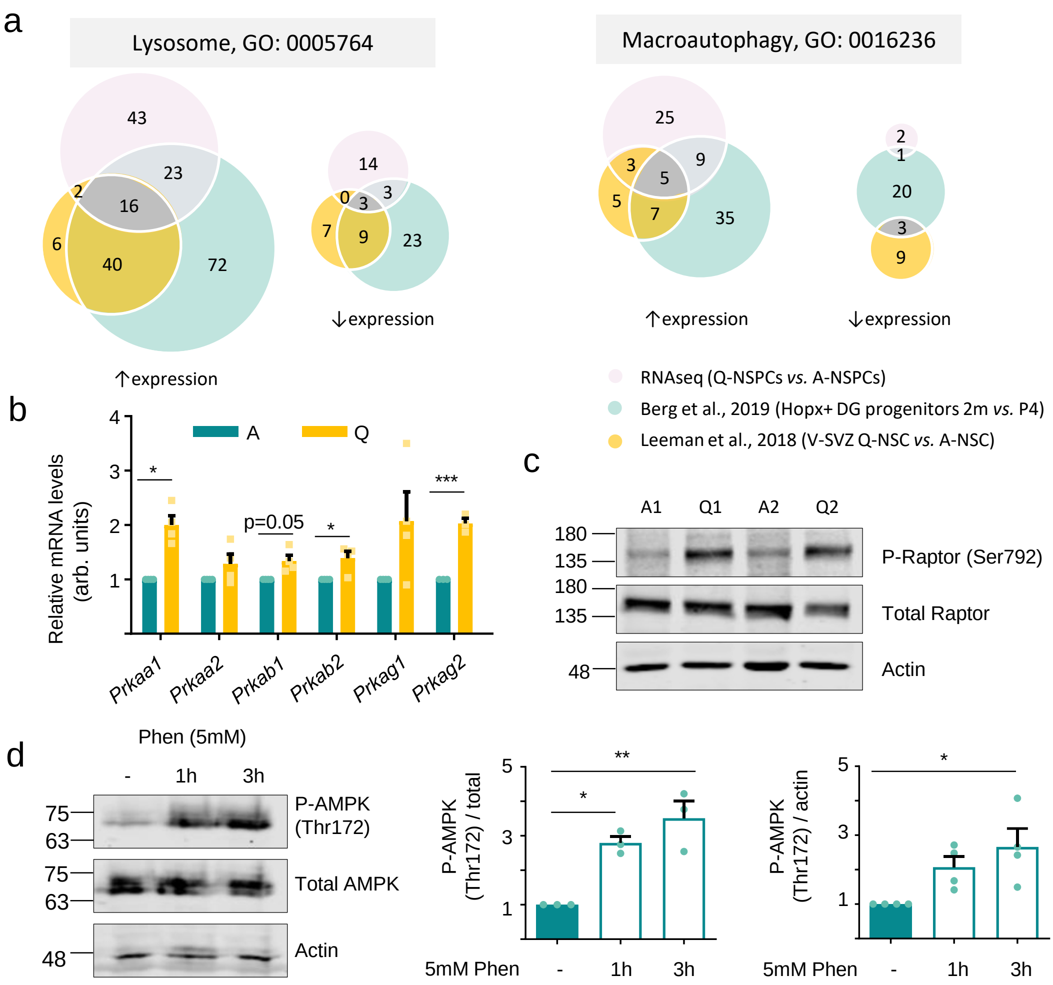

Supplementary Figure 6. Expression of ALP-related genes and Phenphormin assays. a. Venn diagrams identify commons genes associated to Lysosome GO term (Left) and Macroautophagy GO term (Right) between the RNAseq data from this study, Berg et al., 2019 and Leeman et al., 2018's datasets. **b.** Relative expression of AMPK subunits genes in A-NSPCs and Q-NSPCs. Data are represented as mean ± SEM from ≥3 cultures. Statistics: one-sample t-test. **c.** Representative immunoblots of Raptor and P-Raptor (Ser792) in A-NSPCs and Q-NSPCs. β-Actin was used as a loading control. **d.** (Left) Representative immunoblots of AMPK and P-AMPK (Thr172) in A-NSPCs treated with Phenformin (Phen) during 1 or 3h. β-Actin was used as a loading control. (Right) Quantification of activation of AMPK (P-AMPK Thr172) in A-NSPCs after Phenformin treatment. The experiment was repeated 3 (left) or 4 (right) times with similar results. Data are represented as mean ± SEM from n=3 or n=4 cultures (Fig. d). Statistics: 1-way ANOVA. p-value: *<0.05; **<0.001. Source data and all blots are provided as a Source Data file. A, Active NSPCs. Q, Quiescent NSPCs. Phen (Phenformin).

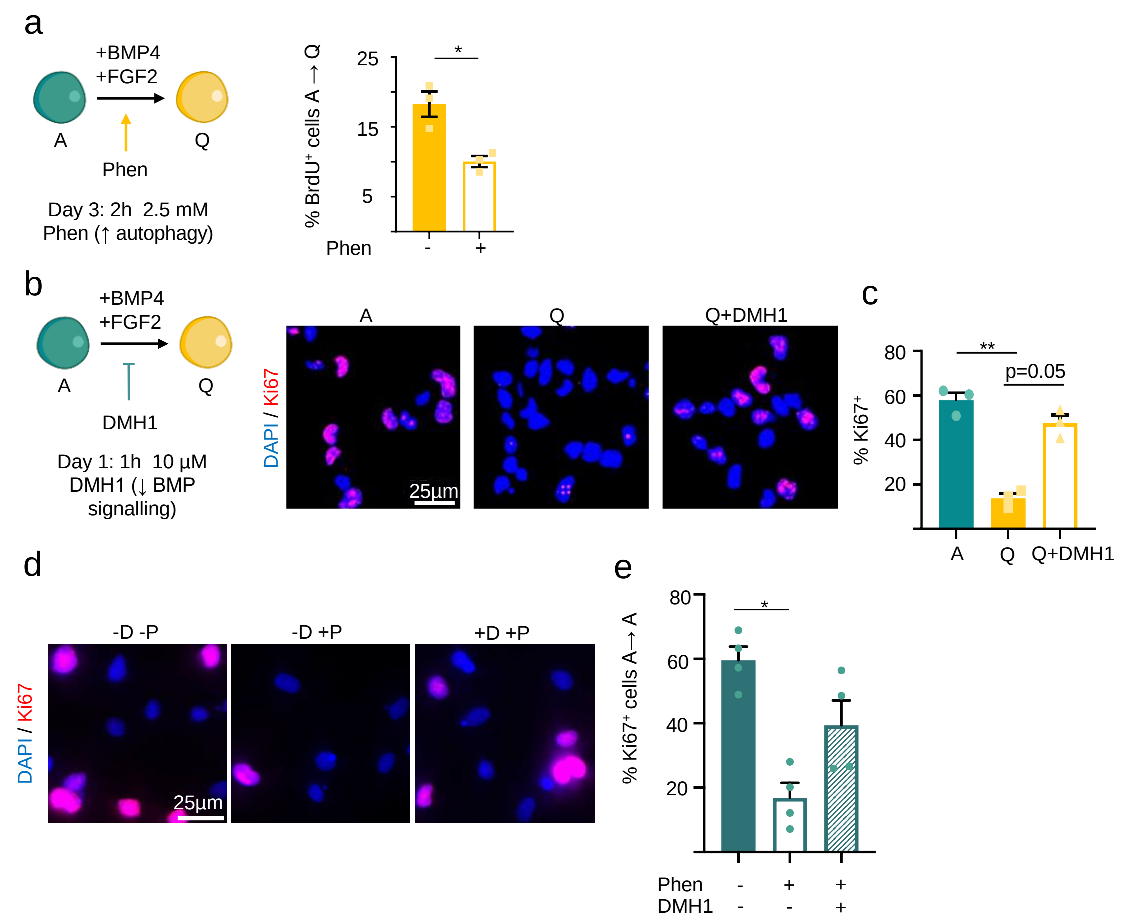

Supplementary Figure 7. BMP type I receptor inhibition rescues the acquisition of NSPC quiescence induced by BMP4 but not by Phen. a. (Left) Functional assay scheme. Phenformin (Phen) activates the autophagy–lysosome pathway. A-NSPCs were treated for 2 h with Phen on Day 3 of quiescence induction. (Right) Percentage of BrdU^{+} cells 24 h after the 2 h Phen treatment of A-NSPCs entering quiescence. Statistics: paired t-test. **b.** Representative immunofluorescence confocal images of Ki67 label (red) in A-NSPC cultures treated with or without DMH1 (10 µM, 1h), followed by quiescence induction with BMP4. Scale bar: 25 µm. **c.** Percentage of Ki67^{+} cells in A-NSPCs treated with or without DMH1 and its subsequent quiescence induction. The experiment was repeated 3 times with similar results. Statistics: RM one-way ANOVA. **d.** Representative immunofluorescence confocal images of Ki67 label (red) in A-NSPC cultures treated with or without DMH1 (10 µM, 1h) and Phen (2.5 mM, 2h). Scale bar: 25 µm. **e.** Percentage of Ki67^{+} cells in A-NSPCs treated with or without DMH1 and Phen. The experiment was repeated 4 times with similar results. Statistics: RM one-way ANOVA. Data are represented as mean ± SEM from n=3 (Fig. a, c) or n=4 (Fig. e) cultures. At least 15 cells were analyzed per experiment and condition. p-value: *<0.05; **<0.001. Representation shown in a. and b. was created with BioRender.com. Source data are provided as a Source Data file. A, Active NSPCs. Q, Quiescent NSPCs. Phen (Phenformin). DMH1 (Dorsomorphin Homolog 1). D, DMH1. P, Phen.

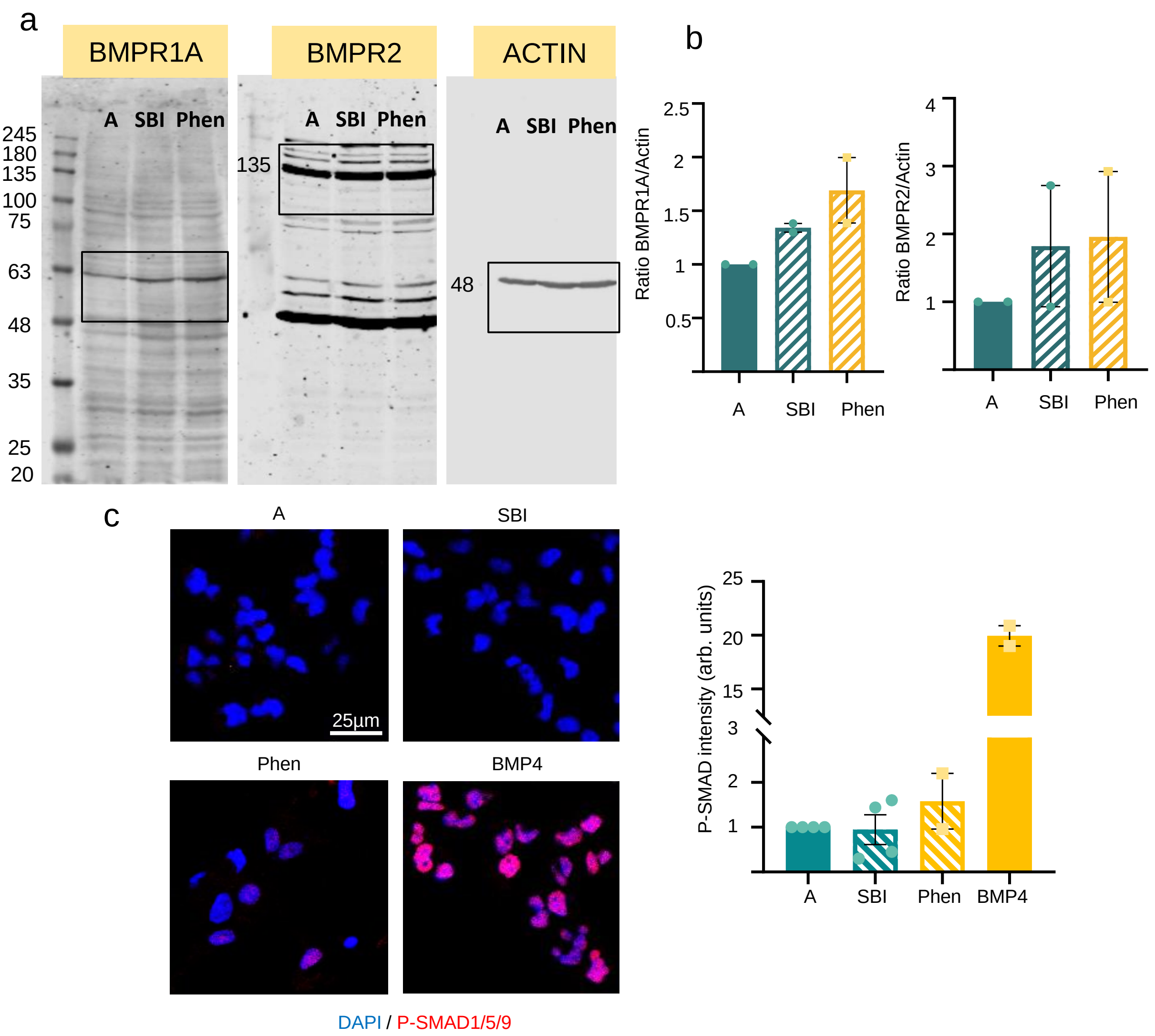

Supplementary Figure 8. BMP type I and II levels and canonical BMP signaling in Phen and SBI treated NSPCs . a. Representative Western immunoblots of BMPRII, BMPR1A and Actin in A-NSPCs treated for 1 h with SBI and 2 h with Phen. **b.** Quantification of the BMPRII and BMPR1A signal using β-Actin levels as a loading control. The experiment was repeated 2 times with similar results. **c.** (Left) Representative immunofluorescence confocal images of P-SMAD1/5/9 nuclear signal (red) in A-NSPC cultures treated with SBI, Phen or BMP4. Scale bar: 25 µm. (Right) Quantification of P-SMAD1/5/9 protein level in the nucleus of A-NSPCs treated with the previously described components, measured as fluorescence intensity. Data are represented as mean ± SEM from n=2 (Fig. b and Fig c: Phen, BMP4) or n=4 (Fig. c: A, SBI) cultures. At least 15 cells were analyzed per experiment and condition. Source data and all blots are provided as a Source Data file. A, Active NSPCs. Phen, Phenformin.

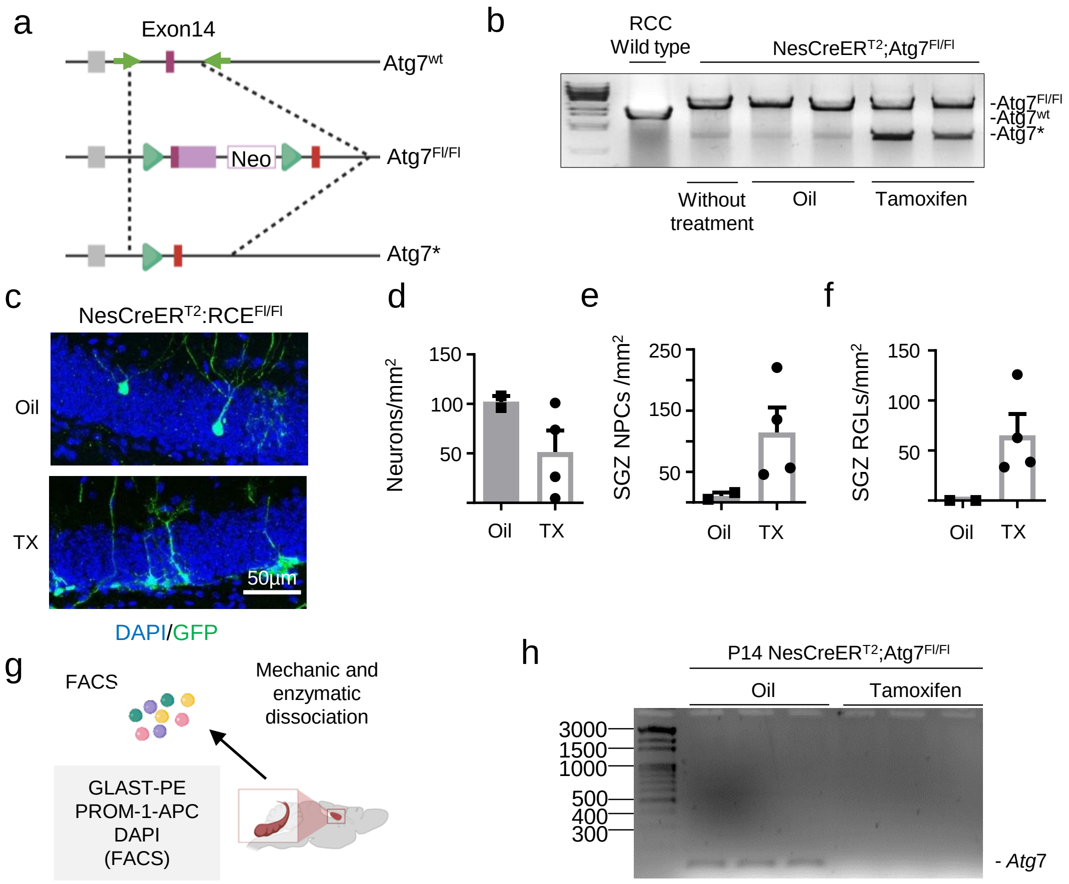

Supplementary Figure 9. Validation of Atg7 allele recombination and loss of Atg7 expression in radial glial-like NSCs of tamoxifen-treated *NesCreER^{T2};Atg7$^{Fl/Fl}$* mice. a. Schematic representation of the targeted allele of *Atg7* gene. Green triangles indicate loxP sequence. Atg7* represent *Atg7* allele after tamoxifen treatment. Orange arrows indicate position of primers used to corroborate recombination. **b.** Validation of recombination in dentate gyrus after tamoxifen treatment by PCR. **c.** 2 months-old-mice *NesCreERT2;RCE^{FL}* were treated with 2mg/day tamoxifen (TX) during 5 days to express GFP in NSCs. Representative immunohistochemistry confocal images of GFP staining in the dentate gyrus of *NesCreERT2;RCE^{FL}* 12 days after Tamoxifen treatment. Scale bar: 50 µm. **d, e, f.** Quantification of GFP$^+$, neurons, GFP$^+$ SGZ NPCs and GFP$^+$ RGLs in *NesCreERT2;RCE^{FL}*. Data are represented as mean ± SEM from n=2 and n=4 Oil-treated and TX-treated mice, respectively. Statistical analysis was not performed since only n=2 Oil-treated mice were counted. **g.** Schematic representation of NSCs isolation by FACS from postnatal 14 (P14) day old *NesCreER^{T2};Atg7$^{Fl/Fl}$* mice hippocampi using GLAST-PE and PROM-1-APC antibodies. **h.** Agarose gel electrophoresis of RT-qPCR reaction, showing *Atg7* expression in GLAST$^+$PROM-1$^+$ cells isolated from P14 *NesCreER^{T2};Atg7$^{Fl/Fl}$* mice hippocampus treated with oil/tamoxifen (3 mice per group). Atg7 ablation was confirmed in tamoxifen treated mice compared to oil animals. Representations shown in a. and g. were created with BioRender.com. Source data are provided as a Source Data file. FACS, Fluorescent Activated Cell Sorter. P14, Postnatal day 14.

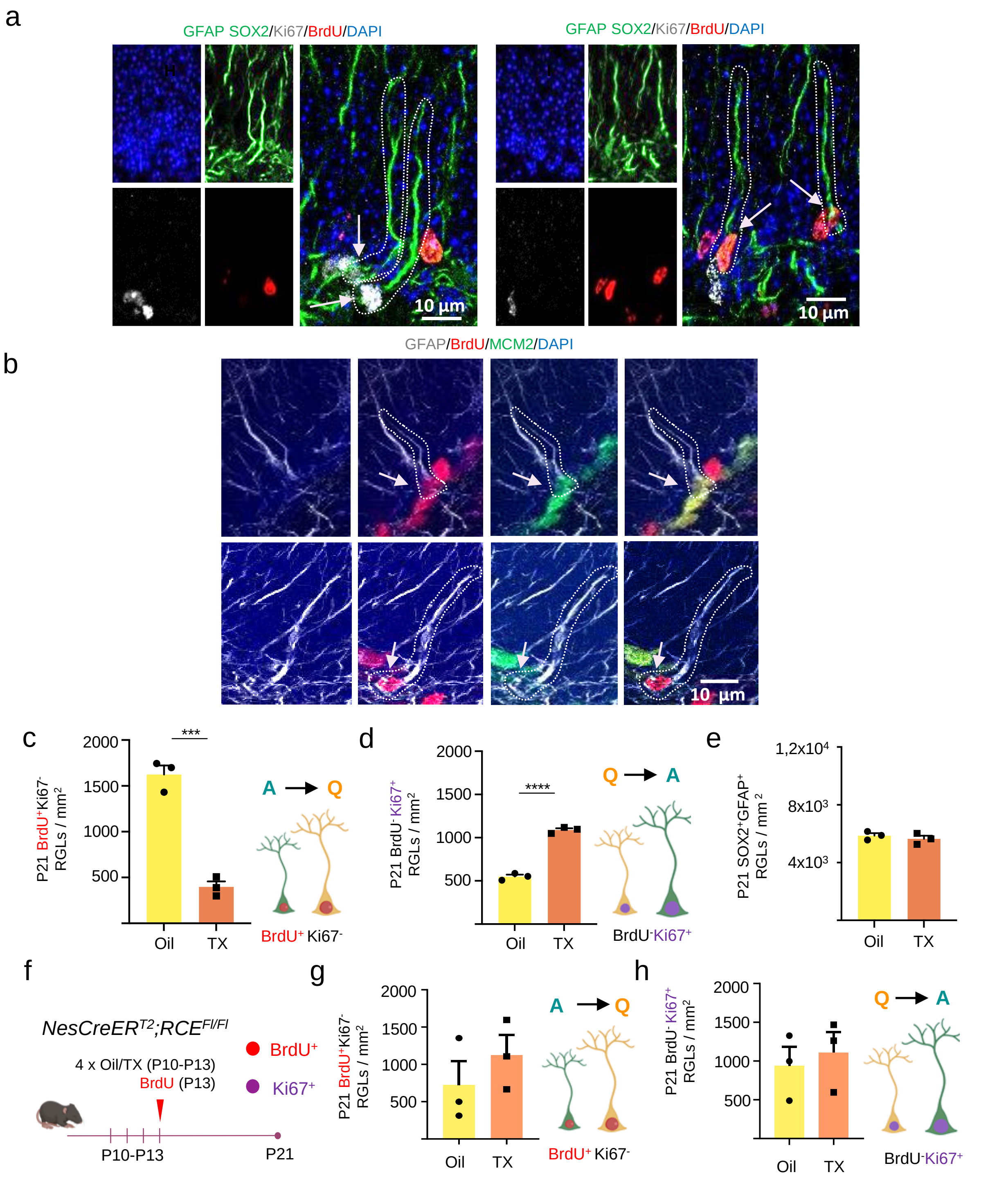

Supplementary Figure 10. RGL analysis in the postnatal SGZ niche and validation of the lack of effect of tamoxifen treatment in *NesCreER*^{T2}*;Atg7*$^{Fl/Fl}$ mice. a. Representative immunohistochemistry confocal images of RGLs labelled for GFAP/SOX2 (green), Ki67 (white) and BrdU (red). Ki67^{+}BrdU^{-} cells (left) and Ki67^{-}BrdU^{+} cells (right) are shown. Scale bar: 10 µm. **b.** Representative immunohistochemistry confocal images of RGLs labelled for MCM2 (green), GFAP (white) and BrdU (red). MCM2^{+}BrdU^{+} cells (above) and MCM2^{-}BrdU^{+} cells (bellow) are shown. Scale bar: 10 µm. **c.** Quantification of active RGLs entering quiescence (BrdU+Ki-67− RGLs/mm2) in the SGZ of *Atg7* conditional knockout (cKO; TX) and control (Oil) P21 mice. Data correspond to independent *in vivo* TX and Oil treatments performed in different mouse litters (n=3 mice per group) compared to Figure 5. **d.** Quantification of quiescent RGLs that become active (BrdU−Ki-67+ RGLs/mm2) in the SGZ of *Atg7* cKO (TX) and control (Oil) P21 mice. **e.** Quantification of total RGLs (SOX2+GFAP+ RGLs/mm^{2}) at P21. **f.** Schematic diagram of the experimental design. A postnatal cohort (P10) of *NesCreERT2;RCE*$^{FL/FL}$ mice was injected with 0.25 mg/day tamoxifen (TX) (P10–P13). Oil was used as control. The mice also received 50 mg/kg BrdU injection to mark dividing cells at P13. Animals were sacrificed at P21 and proliferating cells were identified by Ki-67 staining. **g.** Quantification of active RGLs that become quiescent (BrdU^{+}Ki-67^{-} RGLs/mm^{2}) in the SGZ of oil *vs.* tamoxifen treated animals. The experiments of all panels were repeated 3 times with similar results. Data are presented as mean ± SEM from n=3 mice. Statistics: unpaired t-test. **h.** Quantification of quiescent RGLs that become active (BrdU^{-}Ki-67^{+} RGLs/mm2) in the SGZ of oil *vs.* tamoxifen treated animals. Data are presented as mean ± SEM from n=3 mice. Statistics: unpaired t-test. p-value: ***<0.001; ****<0.0001. Representation shown in c. d. f. g. and h. was created with BioRender.com. Source data are provided as a Source Data file. TX, Tamoxifen. P10, Postnatal day 10. P13, Postnatal day 13. P21, Postnatal day 21.
