# Supplementary material for: Autophagy drives the conversion of developmental neural stem cells to the adult quiescent state
Source: Nat Commun. 2023 Nov 24;14:7541. doi: 10.1038/s41467-023-43222-1 (PMC10673888; doi:10.1038/s41467-023-43222-1)
Supplement: Supplementary file 3 — Description of Additional Supplementary Files [file 41467_2023_43222_MOESM3_ESM.pdf]

Description of Additional Supplementary Files

Title: Supplementary Data 1

Description: This file contains data for the genes genes ascribed to the Macroautophagy and Lysosome Gene Ontology (GO) biological functions significantly up-regulated (UP) or down-regulated (DOWN) in quiescent NSPCs (Padj<0.05) compared to active NSPCs.

Title: Supplementary Data 2

Description: Primer sequences for the rat genes validated by RT-qPCR. FW, forward primer. RV, reverse primer.
