# Supplementary material for: Autophagy drives the conversion of developmental neural stem cells to the adult quiescent state
Source: Nat Commun. 2023 Nov 24;14:7541. doi: 10.1038/s41467-023-43222-1 (PMC10673888; doi:10.1038/s41467-023-43222-1)
Supplement: Supplementary file 6 — Reporting Summary [file 41467_2023_43222_MOESM6_ESM.pdf]

nature portfolio

Corresponding author(s): Helena Mira

Last updated by author(s): Oct 31, 2023

Reporting Summary

Nature Portfolio wishes to improve the reproducibility of the work that we publish. This form provides structure for consistency and transparency in reporting. For further information on Nature Portfolio policies, see our Editorial Policies and the Editorial Policy Checklist.

Statistics

For all statistical analyses, confirm that the following items are present in the figure legend, table legend, main text, or Methods section.

n/a	Confirmed	
☐	☒	The exact sample size (*n*) for each experimental group/condition, given as a discrete number and unit of measurement
☐	☒	A statement on whether measurements were taken from distinct samples or whether the same sample was measured repeatedly
☐	☒	The statistical test(s) used AND whether they are one- or two-sided<br>*Only common tests should be described solely by name; describe more complex techniques in the Methods section.*
☒	☐	A description of all covariates tested
☐	☒	A description of any assumptions or corrections, such as tests of normality and adjustment for multiple comparisons
☐	☒	A full description of the statistical parameters including central tendency (e.g. means) or other basic estimates (e.g. regression coefficient) AND variation (e.g. standard deviation) or associated estimates of uncertainty (e.g. confidence intervals)
☐	☒	For null hypothesis testing, the test statistic (e.g. *F*, *t*, *r*) with confidence intervals, effect sizes, degrees of freedom and *P* value noted<br>*Give P values as exact values whenever suitable.*
☒	☐	For Bayesian analysis, information on the choice of priors and Markov chain Monte Carlo settings
☒	☐	For hierarchical and complex designs, identification of the appropriate level for tests and full reporting of outcomes
☒	☐	Estimates of effect sizes (e.g. Cohen's *d*, Pearson's *r*), indicating how they were calculated

Our web collection on statistics for biologists contains articles on many of the points above.

Software and code

Policy information about availability of computer code

Data collection	For microscopy data collection, we used Confocal microscope Leica SP8. For FACs data colletion, we used BD FACSDiva (version 8.0.1). For qPCR data collection, we used Quant Studio Design and Analysis software (version 1.4.2).
Data analysis	For microscopy image analysis we used Image J-Fiji (1.53q). For FACS analysis, we used FlowJo (version 10.1). For RNAseq analysis, HISAT2 was used to map RNAseq reads to the rat reference genome. Gene expression quantification was performed with HTSeq. DESeq2 package was used to determine differentially expressed genes. Data were represented as heat maps with Excel. GraphPad 8 was used for Statistics.

For manuscripts utilizing custom algorithms or software that are central to the research but not yet described in published literature, software must be made available to editors and reviewers. We strongly encourage code deposition in a community repository (e.g. GitHub). See the Nature Portfolio guidelines for submitting code & software for further information.

Data

Policy information about availability of data

All manuscripts must include a data availability statement. This statement should provide the following information, where applicable:

- Accession codes, unique identifiers, or web links for publicly available datasets
- A description of any restrictions on data availability
- For clinical datasets or third party data, please ensure that the statement adheres to our policy

The RNAseq data generated in this study have been deposited in the NCBI BioProject and BioSample databases under accession code Bioproject PRJNA666074 [https://www.ncbi.nlm.nih.gov/bioproject/PRJNA666074]. The Sequence Read Archive (SRA) experiments with the following accession number were used:

SRX9200622 (https://www.ncbi.nlm.nih.gov/sra/SRX9200622[accn]), SRX9200623 (https://www.ncbi.nlm.nih.gov/sra/SRX9200623[accn]), SRX9200624 (https://www.ncbi.nlm.nih.gov/sra/SRX9200624[accn]), SRX9200625 (https://www.ncbi.nlm.nih.gov/sra/SRX9200625[accn]), SRX9200634 (https://www.ncbi.nlm.nih.gov/sra/SRX9200634[accn]), SRX9200642 (https://www.ncbi.nlm.nih.gov/sra/SRX9200642[accn]). The Source data generated in this study are provided with this paper as a Source Data file.

Human research participants

Policy information about studies involving human research participants and Sex and Gender in Research.

Reporting on sex and gender	No human research was performed.
Population characteristics	No human research was performed.
Recruitment	No human research was performed.
Ethics oversight	No human research was performed.

Note that full information on the approval of the study protocol must also be provided in the manuscript.

Field-specific reporting

Please select the one below that is the best fit for your research. If you are not sure, read the appropriate sections before making your selection.

☒ Life sciences ☐ Behavioural & social sciences ☐ Ecological, evolutionary & environmental sciences

For a reference copy of the document with all sections, see nature.com/documents/nr-reporting-summary-flat.pdf

Life sciences study design

All studies must disclose on these points even when the disclosure is negative.

Sample size	For the in vitro experiments, sample sizes (n) were not predetermined but are similar to those reported in previous publications (Mira et al. Cell Stem Cell 7, 78–89 (2010)). For the in vivo experiments G*Power calculations were employed to reduce the number of animals needed. Sufficient sample size was maintained to obtain a Type I error as low as 0.05 and a power as high as 0.8. For all in vitro determinations, the sample size was n ≥3. For all in vivo determinations, the number of mice that were analysed was n ≥3, except for Extended Data Figure 9. The exact sample size for each analysis is given as a discrete number in the Statistics file.
Data exclusions	Data were not excluded from relevant analysis unless GraphPad Prism identified outliers for normal distributions.
Replication	All in vitro experiments were independently repeated at least 3 times. The number of repetitions are listed in each figure. For the in vivo experiments, independent Oil and tamoxifen treatments of littermates were performed once at P3-P5 (for the NesCreERT2;Atg7Fl/Fl animals sacrificed at P14), twice at P10-P13 (for the animals NesCreERT2;Atg7Fl/Fl sacrificed at P21), once at P10-P13 (for the control animals NesCreERT2;RCE sacrificed at P21) and once at 2 months for the NesCreERT2;RCE and NesCreERT2;Atg7Fl/Fl animals included in Supplementary Figure 9.
Randomization	The order of samples to perform experiments was randomized for each experiment. Animals were randomly assigned to treatment groups.
Blinding	All in vivo experiments were performed blindly with all animals coded so as not to know which treatment each one was receiving. In vitro experiments were not blinded; however, each experiment was paired with appropriate controls and samples were collected and analyzed under identical conditions. RNAseq was performed by a third party at Multigenic Analysis Unit from the UCIM-INCLIVA (University of Valencia, Valencia, Spain) and the samples were numbered to mask identity.

Reporting for specific materials, systems and methods

We require information from authors about some types of materials, experimental systems and methods used in many studies. Here, indicate whether each material, system or method listed is relevant to your study. If you are not sure if a list item applies to your research, read the appropriate section before selecting a response.

Materials & experimental systems

n/a	Involved in the study
	☒ Antibodies
	☒ Eukaryotic cell lines
☒	Palaeontology and archaeology
	☒ Animals and other organisms
☒	Clinical data
☒	Dual use research of concern

Methods

n/a	Involved in the study
☒	ChIP-seq
	☒ Flow cytometry
☒	MRI-based neuroimaging

Antibodies

Antibodies used

Primary antibodies: Ki67 (1:150, Abcam, ab15580), Lamp2 (1:50, Biolegend, 108511), p62 (1:150, Abcam, ab56416), SOX2 (1:250, Gene Tex, GTX101507 and R&D, AF2018), GLAST biotin (1:50, Miltenyi, 130-119-161), Tax1bp1 (1:250, Novus, NBP3-15794), GFP (1:200, Aves-Lab, GFP-1010), GFAP (1:300, Sigma, G3893), BrdU (1:300, Abcam, ab6326), MCM2 (1:150, BD Biosciences, 610700), LC3 (1:1000, Santa Cruz, sc-376404), P-AMPK (1:1000, Cell Signaling, 2535), total AMPK (1:1000, Cell Signaling, 2793), P-ULK (1:1000, Cell Signaling, 5869), total ULK (1:1000, Cell Signaling, 8054), P-Raptor (1:1000, Cell Signaling, 2083), total-Raptor (1:1000, Cell Signaling, 2280), LC3B (1:150, ABclonal, A19665), Phospho-SMAD1 (Ser463/465)/ SMAD5 (Ser463/465)/ SMAD9 (Ser465/467) (D5B10) (1:500, Cell Signaling, #13820), BMPR2 (1:500, Abcam, ab130206), BMPR1A (1:500, Abcam, ab264043) and Actin (1:5000, Sigma-Aldrich, A5441).
Secondary antibodies: Alexa Fluor 555 goat anti-mouse (1:500, Invitrogen, A31570), Alexa Fluor 633 goat anti-mouse (1:100, Invitrogen, A21052), Alexa Fluor 488 goat anti-mouse (1:500, Invitrogen, A21202), Alexa Fluor 488 donkey anti-rabbit (1:500, Invitrogen, A21206), Alexa Fluor 647 donkey anti-rabbit (1:500, Invitrogen, A31573), Cy3 donkey anti-rabbit (1:500, Jackson, 711-165-152), Alexa Fluor 555 goat anti-rat (1:500, Invitrogen, A21434), Alexa Fluor 488 donkey anti-chicken (1:500, Jackson, 703-546-155), Cy2 Streptadivin (1:200, Invitrogen, 016-220-084), IRDye 680LT anti-mouse (1:5000, Licor, 925-68020) and IRDye 800CW anti-rabbit (1:5000, Licor, 925-32211).

Coupled antibodies: GLAST (ACSA-1)-PE (1:50, Miltenyi, 130-118-483) and Anti-Prominin-1-APC (1:10, Miltenyi, 130-102-197).

Validation

Antibodies meet all of the quality control standards defined by manufactures. Validation statements for the commercial antibodies are available on the manufactures websites:

https://www.abcam.com/ki67-antibody-ab15580.html
https://www.biolegend.com/de-at/products/alexa-fluor-647-anti-mouse-cd107b-mac-3-antibody-3274
https://www.abcam.com/sqstm1--p62-antibody-2c11-bsa-and-azide-free-ab56416.html
https://www.genetex.com/Product/Detail/SOX2-antibody-N1C3/GTX101507
https://www.rndsystems.com/products/human-mouse-rat-sox2-antibody_af2018
https://www.miltenyibiotec.com/ES-en/products/glast-acsa-1-antibody-anti-human-mouse-rat-acsa-1.html#gref
https://www.aveslabs.com/products/anti-green-fluorescent-protein-antibody-gfp
https://www.sigmaaldrich.com/ES/es/product/sigma/g3893
https://www.abcam.com/brdu-antibody-bu175-icr1-proliferation-marker-ab6326.html
https://www.bdbiosciences.com/en-us/products/reagents/microscopy-imaging-reagents/immunofluorescence-reagents/Purified-Mouse-Anti-BM28.610700
https://www.scbt.com/es/p/map-lc3beta-antibody-g-9
https://www.cellsignal.com/products/primary-antibodies/phospho-ampka-thr172-40h9-rabbit-mab/2535?_requestid=5642846
https://www.cellsignal.com/products/primary-antibodies/ampka-f6-mouse-mab/2793
https://www.cellsignal.com/products/primary-antibodies/phospho-ulk1-ser555-d1h4-rabbit-mab/5869
https://www.cellsignal.com/products/primary-antibodies/ulk1-d8h5-rabbit-mab/8054
https://www.cellsignal.com/products/primary-antibodies/phospho-raptor-ser792-antibody/2083
https://www.cellsignal.com/products/primary-antibodies/raptor-24c12-rabbit-mab/2280
https://abclonal.com/catalog-antibodies/[KOValidated]LC3BRabbitmAb/A19665
https://www.cellsignal.com/products/primary-antibodies/phospho-smad1-ser463-465-smad5-ser463-465-smad9-ser465-467-d5b10-rabbit-mab/13820
https://www.abcam.com/products/primary-antibodies/bmpr2-antibody-1f12-ab130206.html
https://www.abcam.com/products/primary-antibodies/bmpr1a-antibody-ab264043.html
https://www.sigmaaldrich.com/ES/es/product/sigma/a5441
https://www.miltenyibiotec.com/ES-en/products/glast-acsa-1-antibody-anti-human-mouse-rat-acsa-1.html?countryRedirected=1#pe:30-tests-in-60-ul
https://www.miltenyibiotec.com/ES-en/products/prominin-1-antibody-anti-mouse-mb9-3g8.html#apc:30-ug-in-200-ul
https://www.novusbio.com/products/tax1bp1-antibody-7y9w3_nbp3-15794

Eukaryotic cell lines

Policy information about cell lines and Sex and Gender in Research

Cell line source(s)

No commercial or banked cell lines were used. Primary mouse hippocampal neural stem cells from P3, P14 and P21 mice were isolated as described in the Methods section. Rat adult hippocampal neural stem and progenitor cells were stocks from

	Mira et al. Cell Stem Cell 7, 78–89 (2010).
Authentication	No commercial or banked cell lines were used.
Mycoplasma contamination	All lines were tested negative for mycoplasma.
Commonly misidentified lines (See ICLAC register)	No commonly misidentified lines were used.

Animals and other research organisms

Policy information about studies involving animals; ARRIVE guidelines recommended for reporting animal research, and Sex and Gender in Research

Laboratory animals	Wild type C57BL/6JRccHsd mice were purchased from Inotiv (https://www.inotivco.com/model/c57bl-6jrcchsd). Transgenic C57BL/6-Tg(Nes-cre/ERT2)4Imayo (NesCreERT2) were described in Imayoshi et al. Genesis 44, 233–238 (2006). B6.Cg-ATG7tm1Tchi (Atg7Fl/Fl) were described in Komatsu et al. J. Cell Biol. 169, 425–434 (2005). RCE mice were obtained from Jackson Laboratory (Bar Harbor, ME) (STOCK Gt(ROSA)26Sortm1.1(CAG-EGFP)Fsh/Mmjax (#32037)). NesCreERT2 mice were crossed with Atg7Fl/Fl or RCE mice to obtain NesCreERT2;Atg7Fl/Fl and NesCreERT2;RCE.<br>For experiments with wild type animals, postnatal (P3 to P21) C57BL/6JRccHsd male and female mice were used. For experiments with transgenic animals, postnatal (P3 to P21) NesCreERT2;Atg7Fl/Fl male and female mice were used. For control experiments, 21 days old NesCreERT2;RCE male and female mice, and 2 months old NesCreERT2;RCE and NesCreERT2;Atg7Fl/Fl male mice were used. Animals from the same litter were randomly assigned to the Tamoxifen or Oil group.
Wild animals	No wild animals were used for this study.
Reporting on sex	Sex was not considered in the study design and analysis. Young postnatal males and females were pooled for this study. Animals from the same litter were randomly assigned to the Tamoxifen or Oil group. Data cannot be disaggregated for sex. For some control experiments, provided as Supplementary Information, 2 month old male mice were used.
Field-collected samples	No field-collected samples were used for this study.
Ethics oversight	All experimental procedures were approved by the Institutional Animal Care and Use Committee of Instituto de Biomedicina de Valencia and by the Bioethical Comittee of CSIC (protocol 2018/VSC/PEA/0053 and protocol 1458/2023).

Note that full information on the approval of the study protocol must also be provided in the manuscript.

Flow Cytometry

Plots

Confirm that:

- [x] The axis labels state the marker and fluorochrome used (e.g. CD4-FITC).
- [x] The axis scales are clearly visible. Include numbers along axes only for bottom left plot of group (a 'group' is an analysis of identical markers).
- [x] All plots are contour plots with outliers or pseudocolor plots.
- [x] A numerical value for number of cells or percentage (with statistics) is provided.

Methodology

Sample preparation	Decapitation (P3 mice) and cervical dislocation (P21 mice) were used as euthanasia methods in MACS and FACS procedures. Hippocampi from postnatal day 3 or day 21 C57BL/6JRccHsd mice were dissociated using Neural Tissue Dissociation Kit (Miltenyi, 130-092-628) with gentle MACS Octo Dissociator (Miltenyi, 130-095-937). Hippocampal NSCs were sorted by flow cytometry based on staining signals. Cells were labelled with GLAST (ACSA-1)-PE (1:50, Miltenyi, 130-118-483) and Anti-Prominin-1-APC (1:10, Miltenyi, 130-102-197) antibodies.<br><br>For hippocampal NSCs isolation of P14 NesCreERT2;Atg7Fl/Fl mice treated with oil/tamoxifen, cells were labelled with GLAST (ACSA-1)-PE (1:50, Miltenyi, 130-118-483) and Anti-Prominin-1-APC (1:10, Miltenyi, 130-102-197) antibodies.
Instrument	BD FACSAria™ III Cell Sorter.
Software	BD FACSDiva (version 8.0.1) and FlowJo (version 10.1).
Cell population abundance	NSCs sorted from P3 and P21 mice were 2.16 ± 0.53 and 0.54 ± 0.13% (mean ± SEM), respectively, of the total cells analyzed. On the other hand, NSCs sorted from oil or tamoxifen treated P14 mice were 0.93 and 0.33%, respectively, of the total cells analyzed.

Gating strategy	Forward scatter/side scatter gatings were used to remove doublets and debris. DAPI negative cells were considered viable. Compensations were done on single-color controls and gates were set on unstained samples. Hippocampal NSCs were sorted based on the double staining (GLAST-PE+/PROM-1-APC+).

☒ Tick this box to confirm that a figure exemplifying the gating strategy is provided in the Supplementary Information.
